# RETRACTION: Analyzing Cross‐Talk of EPO and EGF Genes Along With Evaluating Therapeutic Potential of *Cinnamomum Verum* in Cigarette‐Smoke‐Induced Lung Pathophysiology in Rat Model

**DOI:** 10.1002/fsn3.70031

**Published:** 2025-03-18

## Abstract

**RETRACTION**: H. Anwar, S. Navaid, H. Muzaffar, G. Hussain, M. N. Faisal, M. U. Ijaz and S. Riđanović, “Analyzing Cross‐Talk of EPO and EGF Genes Along With Evaluating Therapeutic Potential of *Cinnamomum Verum* in Cigarette‐Smoke‐Induced Lung Pathophysiology in Rat Model,” *Food Science & Nutrition* 11, no. 3 (2023): 1486–1498, https://doi.org/10.1002/fsn3.3188.

The above article, published online on 11 January 2023 in Wiley Online Library (wileyonlinelibrary.com), has been retracted by agreement between the journal Editor‐in‐Chief, Y. Martin Lo; and Wiley Periodicals LLC. The retraction has been agreed following an investigation into concerns raised by a third party. The investigation revealed an overlap between the positive control images for day 14 in Figure 5 and day 21 in Figure 7. The authors acknowledged their error and provided some raw data along with an alternative positive control image for day 14 in Figure 5. However, a further concern was raised regarding an overlap observed between the negative control images for day 14 in Figure 5 and day 18 in Figure 6. This overlap was also evident in the raw images previously supplied by the authors. The authors provided an explanation, but the editors found it unsatisfactory. Consequently, the editors have lost confidence in the data presented and consider the conclusions to be significantly compromised. The authors were informed of the retraction.

**RETRACTION**: H. Anwar, S. Navaid, H. Muzaffar, G. Hussain, M. N. Faisal, M. U. Ijaz and S. Riđanović, “Analyzing Cross‐Talk of EPO and EGF Genes Along With Evaluating Therapeutic Potential of *Cinnamomum Verum* in Cigarette‐Smoke‐Induced Lung Pathophysiology in Rat Model,” *Food Science & Nutrition* 11, no. 3 (2023): 1486–1498, https://doi.org/10.1002/fsn3.3188.

The above article, published online on 11 January 2023 in Wiley Online Library (wileyonlinelibrary.com), has been retracted by agreement between the journal Editor‐in‐Chief, Y. Martin Lo; and Wiley Periodicals LLC. The retraction has been agreed following an investigation into concerns raised by a third party. The investigation revealed an overlap between the positive control images for day 14 in Figure 5 and day 21 in Figure 7. The authors acknowledged their error and provided some raw data along with an alternative positive control image for day 14 in Figure 5. However, a further concern was raised regarding an overlap observed between the negative control images for day 14 in Figure 5 and day 18 in Figure 6. This overlap was also evident in the raw images previously supplied by the authors. The authors provided an explanation, but the editors found it unsatisfactory. Consequently, the editors have lost confidence in the data presented and consider the conclusions to be significantly compromised. The authors were informed of the retraction.

